# Electrogenic Cation Binding in the Electroneutral Na^+^/H^+^ Antiporter of Pyrococcus abyssi*

**DOI:** 10.1074/jbc.M116.761080

**Published:** 2016-11-07

**Authors:** Octavian Călinescu, Mark Linder, David Wöhlert, Özkan Yildiz, Werner Kühlbrandt, Klaus Fendler

**Affiliations:** From the Departments of ‡ Biological Chemistry and; ¶ Structural Biology, Max Planck Institute of Biophysics, 60438 Frankfurt am Main, Germany and; the § Department of Biophysics, “Carol Davila” University of Medicine and Pharmacy, 050474 Bucharest, Romania

**Keywords:** archaea, electrophysiology, membrane transport, pH regulation, sodium-proton exchange, PaNhaP, solid supported membrane

## Abstract

Na^+^/H^+^ antiporters in the CPA1 branch of the cation proton antiporter family drive the electroneutral exchange of H^+^ against Na^+^ ions and ensure pH homeostasis in eukaryotic and prokaryotic organisms. Although their transport cycle is overall electroneutral, specific partial reactions are electrogenic. Here, we present an electrophysiological study of the PaNhaP Na^+^/H^+^ antiporter from *Pyrococcus abyssi* reconstituted into liposomes. Positive transient currents were recorded upon addition of Na^+^ to PaNhaP proteoliposomes, indicating a reaction where positive charge is rapidly displaced into the proteoliposomes with a rate constant of *k* >200 s^−1^. We attribute the recorded currents to an electrogenic reaction that includes Na^+^ binding and possibly occlusion. Subsequently, positive charge is transported out of the cell associated with H^+^ binding, so that the overall reaction is electroneutral. We show that the differences in pH profile and Na^+^ affinity of PaNhaP and the related MjNhaP1 from *Methanocaldococcus jannaschii* can be attributed to an additional negatively charged glutamate residue in PaNhaP. The results are discussed in the context of the physiological function of PaNhaP and other microbial Na^+^/H^+^ exchangers. We propose that both, electroneutral and electrogenic Na^+^/H^+^ antiporters, represent a carefully tuned self-regulatory system, which drives the cytoplasmic pH back to neutral after any deviation.

## Introduction

Electroneutral Na^+^/H^+^ exchangers are responsible for cytosolic pH homeostasis and intracellular volume regulation (1, 2). They are classified in the CPA1 subfamily of the monovalent cation proton antiporter (CPA)^2^ superfamily (3). Human CPAs, in particular the NHE (sodium proton exchanger) transporters, are of major interest because they are implicated in a number of serious pathologies and are important drug targets (1). So far, there is no detailed structural information on eukaryotic CPAs, but the structures of the Na^+^/H^+^ antiporter from *Methanocaldococcus jannaschii* (MjNhaP1) (4) and *Pyrococcus abyssi* (PaNhaP) (5) have recently been determined. With their high sequence homology to human exchangers, especially in the ion-binding region (5), PaNhaP and MjNhaP1 serve as excellent model systems for identifying key residues and elucidating the transport mechanism of NHE transporters.

In a recent study we demonstrated that the competition-based transport mechanism first proposed for the electrogenic CPA2 antiporter NhaA (EcNhaA) from *Escherichia coli* (6) also applies to the electroneutral CPA1 antiporter MjNhaP1 (7). Members of the CPA1 and CPA2 families share similarities in the 6-helix bundle that are essential for transport (4). However, a major difference between members of the two subfamilies is the presence of a conserved Asn-Asp motif in the binding site of the CPA1 exchangers, whereas CPA2 exchangers have a conserved Asp-Asp motif in the same position (4, 5). The additional negatively charged Asp residue in CPA2 is proposed to be responsible for the extra H^+^ that is transported by these exchangers compared with CPA1 (6, 7).

The striking feature of the model, in which H^+^ and Na^+^ ions compete for a common binding site, is that it is self-regulatory, ensuring that transport activity is switched off at extreme pH values to prevent excessive acidification or alkalinization of the cytoplasm (7, 8). For an electrogenic antiporter such as EcNhaA, at least one electrogenic reaction is required per transport cycle. In the case of EcNhaA this reaction was seen to be the Na^+^ translocation step (6). For an electroneutral exchanger like MjNhaP1, an electrogenic reaction is, in theory, not required. However, the study on MjNhaP1 revealed that this exchanger, although overall electroneutral, has at least two electrogenic transport steps, which we assigned to the translocation of the Na^+^ and H^+^ substrate ions across the membrane (7). This enabled us to monitor the activity of the transporter by solid-supported membrane (SSM)-based electrophysiology, an experimental technique that is particularly appropriate for the characterization of prokaryotic membrane transporters (9). The technique is better suited to the investigation of Na^+^/H^+^ exchangers than the more commonly used fluorescence dequenching as it allows better pH control and has an improved dynamic range for the measured activity (10).

As we were able to identify electrogenic reactions in MjNhaP1, even though it is overall electroneutral, we were interested to find out whether they also exist in PaNhaP. Furthermore, because PaNhaP has an extra negatively charged residue (Glu-73) in the substrate binding site that has no homologue in MjNhaP1 (5), we wanted to investigate the role of this residue in the transport mechanism of PaNhaP.

We found that the competition-based transport mechanism that applies to EcNhaA and MjNhaP1 also describes the activity of PaNhaP. As in MjNhaP1, we uncovered electrogenic steps in the transport cycle of PaNhaP that effect the translocation of positive charge, both in the wild-type protein and in a mutant where Glu-73 was replaced by alanine. These electrogenic steps are attributable to the binding and possibly the occlusion of the Na^+^ substrate ion. Our results establish a model of substrate translocation and define the role of Glu-73 in PaNhaP.

## Results

### 

#### 

##### Electrogenic Substrate Translocation in PaNhaP_WT_

**FIGURE 1.**
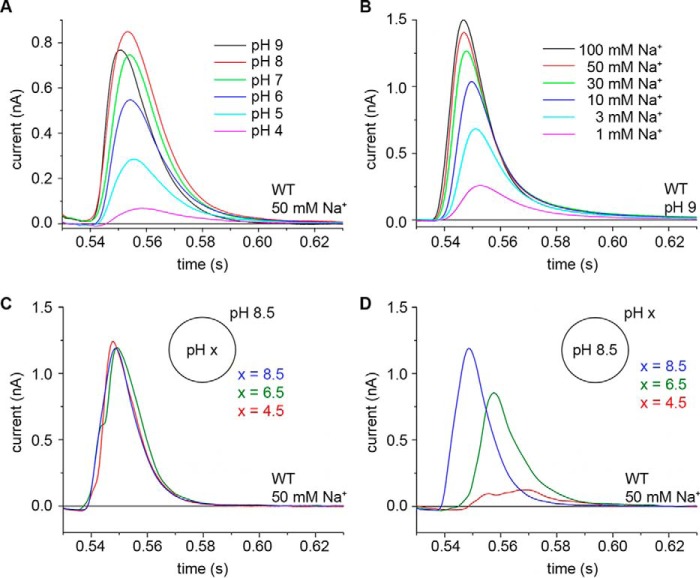
**Transient currents recorded on PaNhaP_WT_ proteoliposomes under different pH conditions.** *A*, symmetrical pH. Transient currents recorded after a 50 mm Na^+^ concentration jump at different pH values. *B*, symmetrical pH. Transient currents recorded after Na^+^ concentration jumps at pH 9. *C* and *D*, asymmetrical pH. Applying a double-solution exchange flow protocol the pH was varied independently inside (*C*) and outside (*D*) the proteoliposomes. Only the outside pH affects current amplitudes. Therefore, the release of the Na^+^ ion is not recorded. Representative results of recordings performed on three individual sensors are shown.

Proteoliposomes containing reconstituted PaNhaP_WT_ were subjected to Na^+^ concentration jumps under conditions of symmetrical or asymmetrical pH (pH inside *versus* outside the proteoliposomes). We found that even though PaNhaP is overall electroneutral, transient currents were measurable in response to Na^+^ concentration jumps (Fig. 1). This suggests an electrogenic Na^+^ translocation step in the transport cycle of PaNhaP, as previously observed for the electroneutral MjNhaP1 and for the electrogenic EcNhaA (6, 7). Furthermore, as in MjNhaP1, the polarity of the recorded transients was positive, indicating that positive charge is displaced inside the proteoliposomes upon Na^+^ binding and/or translocation.

The transient currents are dominated by the time dependence of the solution exchange process on the SSM surface. From the curve of the transient currents (Fig. 1) we estimate a rate constant of *k* > 200 s^−1^ (11). The displaced charge was calculated as 27 ± 5 pC, which is three times larger than in MjNhaP1 (9 ± 2 pC). It is interesting to compare this charge displacement to the value of 96 ± 19 pC estimated for translocation of one elementary charge in lactose permease (12). Although this charge varies to some extent with the sample preparation and the adsorption of proteoliposomes to the SSM, we can conclude that a significant fraction of one elementary charge is displaced upon a saturating Na^+^ concentration jump.

**FIGURE 2.**
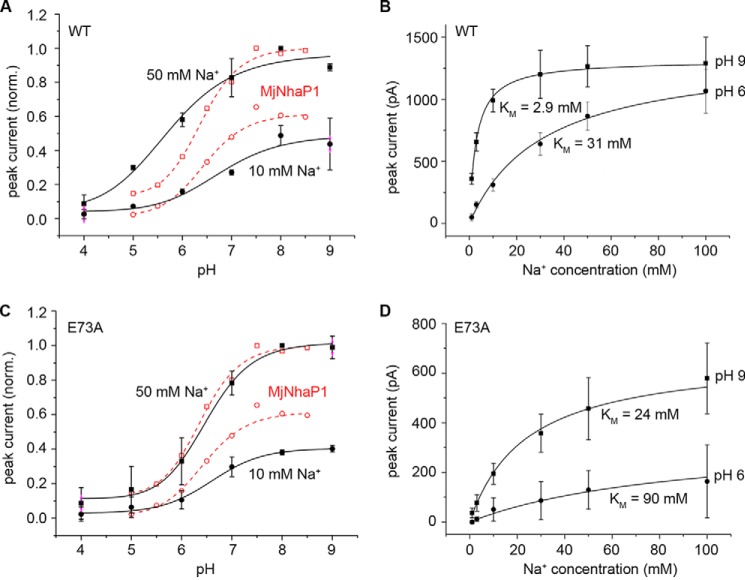
**pH and Na^+^ dependence of recorded currents for PaNhaP_WT_ and PaNhaP_E73A_.** pH dependence of the transient current amplitude determined for PaNhaP_WT_ (*A*) and PaNhaP_E73A_ (*C*) with Na^+^ concentration jumps of 10 (*circles*) and 50 mm (*squares*). Traces for MjNhaP1 (*open circles* and *squares*) are overlaid for comparison. Na^+^ dependence of transient currents recorded on PaNhaP_WT_ (*B*) and PaNhaP_E73A_ (*D*) proteoliposomes at pH 6 and 9. Measurements were performed on three different SSM sensors and are given as average values ± S.D. Currents in *A* and *C* were normalized to the amplitude of a 50 mm Na^+^ concentration jump at pH 8.

Following concentration jumps of 50 mm Na^+^ (Fig. 1*A*), the recorded transient currents increased in amplitude with pH, reaching a plateau at pH 7 and above (Figs. 1*A* and 2*A*). Compared with the pH dependence of MjNhaP1 (Fig. 2*A*) (7) the transients recorded for PaNhaP_WT_ increase more gradually with pH. Note that, as for MjNhaP1 (7), the pH profile of the transient currents does not represent the pH profile of steady-state transport activity, as it only reports on a Na^+^-dependent partial reaction in the transport cycle.

When transient currents were recorded at pH 9 using increasing jumps in Na^+^ concentration, the transient currents increased hyperbolically with the Na^+^ concentration (Figs. 1*A* and 2*B*). The apparent Na^+^ affinity of the transporter decreased with pH, indicating competition between Na^+^ and H^+^ (Fig. 2*B*).

Na^+^ concentration jumps at asymmetrical pH revealed that the current amplitudes were independent of the internal pH, whereas they varied with external pH (Fig. 1, *C* and *D*). This is indicative of an early reaction in the transport cycle associated with Na^+^ binding. In particular, it shows that the currents do not depend on internal H^+^ binding or translocation to the outside.

##### Response of PaNhaP_E73A_ to Na^+^ Concentration Jumps

Positive transient currents were also recorded when PaNhaP_E73A_ was subjected to Na^+^ concentration jumps. Compared with the wild-type, the slope of the pH dependence for PaNhaP_E73A_ was steeper (Fig. 2*C*). Interestingly, the pH dependence of the Na^+^ transients for PaNhaP_E73A_ recorded after saturating (50 mm) Na^+^ concentration jumps was virtually identical to MjNhaP1. Thus, from a functional point of view, the most striking difference between PaNhaP and MjNhaP1 seems to be the presence or absence of Glu-73 in the substrate-binding site. Another effect of replacing Glu-73 by Ala in PaNhaP was a 10-fold reduction in the apparent affinity for Na^+^ at pH 9 in PaNhaP_E73A_, compared with PaNhaP_WT_ (Fig. 2*D*).

##### Na^+^ Exchange in PaNhaP_WT_ Is Absent at Alkaline pH

**FIGURE 3.**
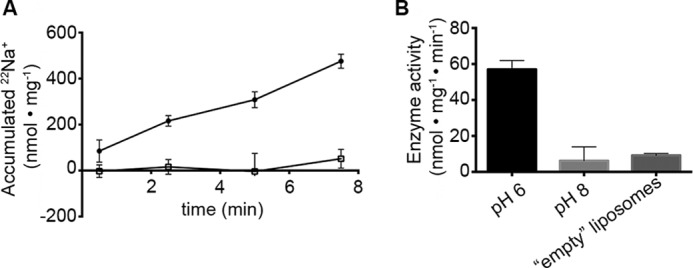
**Na^+^ counterflow activity of PaNhaP.** *A,* accumulation of ^22^Na^+^ as a result of counterflow of sodium at pH 6 (*circles*) is linear in the entire range. Counterflow activity at pH 8 (*open squares*) is not detectable. *B,* the accumulation rate of 57 nmol min^−1^ mg^−1^ at pH 6 corresponds to a turnover of 0.05 s^−1^. Data are shown as average values ± S.D. Each data point corresponds to 3 individual experiments.

To complement our electrophysiological measurements, we assayed ^22^Na^+^ counterflow in WT PaNhaP proteoliposomes at pH 6 and 8 (Fig. 3). Whereas ^22^Na^+^ accumulated at pH 6, there was no ^22^Na^+^ uptake by the proteoliposomes at pH 8, indicating a lack of sodium exchange at elevated pH, where physiological Na^+^/H^+^ antiport by PaNhaP is down-regulated (5).

##### Response of PaNhaP to Tl^+^

The crystal structure of PaNhaP was solved at a resolution of 3.2 Å in the presence of Tl^+^ ions, which were identified in the binding site by their anomalous signal (5). As Tl^+^ was shown to be transported by PaNhaP (5), we asked whether Tl^+^ concentration jumps induce similar transient currents as corresponding changes in Na^+^ concentration.

Following Tl^+^ concentration jumps on WT PaNhaP proteoliposomes, we measured positive transient currents, but with amplitudes comparable with those induced by Tl^+^ concentration jumps performed on “empty” liposomes, devoid of protein (data not shown).

**FIGURE 4.**
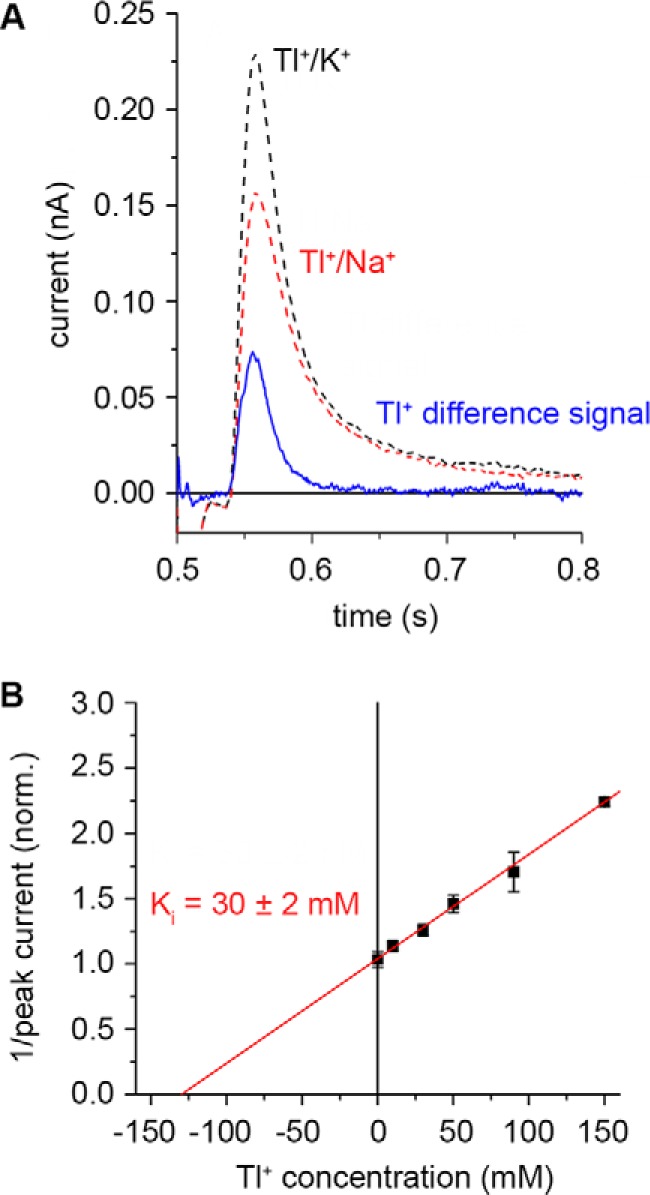
**Effect of Tl^+^ on PaNhaP.** *A*, transient currents recorded after concentration jumps of 30 mm Tl^+^ in the presence or absence of Na^+^ at pH 8. The *blue trace* represents the Tl^+^-dependent transient current. *B*, inhibitory effect of different concentrations of Tl^+^ on the amplitude of transient currents recorded following 10 mm Na^+^ concentration jumps at pH 8. Measurements in *B* were performed on three different SSM sensors and amplitudes of the transient currents were normalized to the value recorded in the absence of Tl^+^. The intersection of the reciprocal plot at the concentration axis is −*K_i_*([Na^+^]/*K_m_+1*). With [Na^+^] = 10 mm and *K_m_* = 2.9 mm, *K_i_* was calculated as given in the figure.

Therefore, to compare measurements performed on the same SSM sensor directly, we subjected PaNhaP_WT_ proteoliposomes to 30 mm Tl^+^ concentration jumps in the presence or absence of 50 mm Na^+^, enough to saturate the transporter with Na^+^. The recorded transients (Fig. 4*A*) were subtracted and the Tl^+^-dependent current was recovered.

We also tested whether the presence of Tl^+^ in the activating and non-activating solutions has any inhibitory effect on Na^+^ translocation. Indeed, Tl^+^ acted as a competitive inhibitor, as it reduced the amplitude of transient currents recorded for 10 mm Na^+^ concentration jumps (Fig. 4*B*) with a *K_i_* of 30 mm at pH 8.

## Discussion

### 

#### 

##### Rapid Na^+^-induced Charge Displacement in PaNhaP

Transient currents after a Na^+^ concentration jump are positive. The size (Table 1) and time dependence of the current is characteristic of a pre-steady state charge displacement, as indicated by the following considerations: 1) the current decays rapidly and its time dependence is virtually independent of the Na^+^ concentration; 2) the current is sensitive only to external pH and insensitive to internal pH. This finding is consistent with CPA1 Na^+^/H^+^ exchangers being electroneutral, as previously observed for MjNhaP1 (7). The reaction that results in the observed transient currents is rapid (*k* > 200 s^−1^) and displaces a significant fraction of one elementary charge.

**TABLE 1:** **Kinetic parameters determined for cation and proton binding to PaNhaP_WT_ and PaNhaP_E73A_** *I*_max_ is the average peak current at optimal substrate concentration. p*K*_app_ is the apparent p*K* and refers to the pH of half-maximal electrogenic activity. Parameters for Tl^+^ binding are from Fig. 4. Data for MjNhaP1 (7) are included for comparison.

	*I*_max_*^a^*	*K_m,alk_**^b^* (Na^+^/Tl^+^)	*K_m,ac_**^c^* (Na^+^)	p*K*_app_
PaNhaP_WT_	1.3 nA	2.9 ± 0.2 mm	31 ± 5 mm	5.7
PaNhaP_E73A_	0.7 nA	24 ± 4 mm	90 ± 60 mm	6.4
PaNhaP_WT_ (Tl^+^)	0.15 nA	30 ± 2 mm		
MjNhaP1	0.7 nA	6.7 mm	30 mm	6.3

*^a^ I*_max_ was determined for 50 mm Na^+^ concentration jumps at pH 8 (PaNhaP) or 7.5 (MjNhaP1).

*^b^ K_m,alk_* was determined for PaNhaP at pH 9 (Na^+^) or pH 8 (Tl^+^), and for MjNhaP1 at pH 7.5.

*^c^ K_m,ac_* was determined at pH 6.

The time and substrate dependence of the positive charge displacement is similar to that in MjNhaP1 (7). In MjNhaP1 this charge displacement was tentatively assigned to Na^+^ binding, followed by the conformational transition and the release of Na^+^ to the liposome interior, in the order C_o_ + Na → C_o_Na → C_i_Na → C_i_ + Na (Fig. 7*A*). However, counterflow experiments with PaNhaP (Fig. 3) show that the complete Na^+^ translocation process as outlined above is slow (∼0.05 s^−1^) and absent at alkaline pH, where our currents are largest. We, therefore, attribute the observed electrogenic reaction to rapid Na^+^ binding. Because the process is electrogenic, simple binding to a surface-exposed Na^+^ binding site is unlikely. Rather, a subsequent conformational change that occludes the substrate ion may be responsible for the major part of the charge displacement. In the following we will refer to this reaction as electrogenic Na^+^ binding.

##### Functional Evidence for Two Carboxylates in the Na^+^ Binding Site of PaNhaP

**FIGURE 5.**
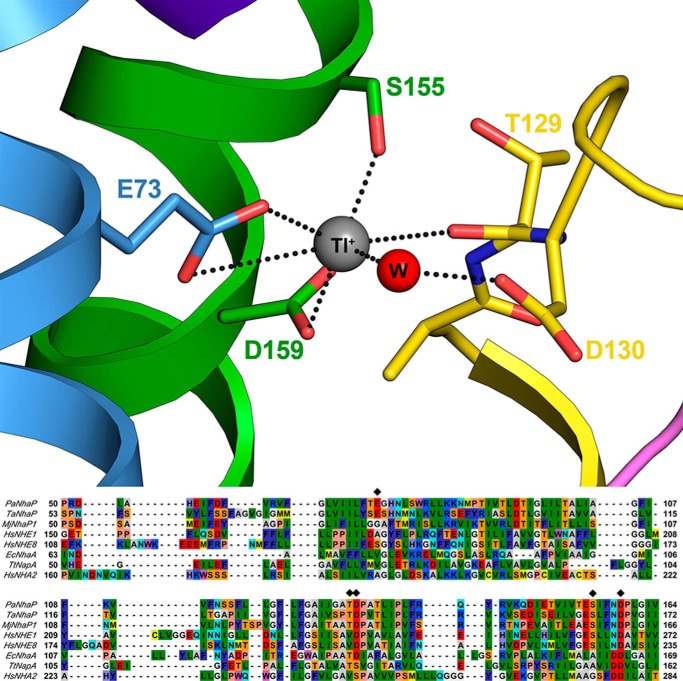
**Structure of the cation-binding site of PaNhaP with bound Tl^+^.** *W* is a water molecule contributing to coordination of the cation. In the *lower panel,* a sequence comparison of the cation-binding site of Na^+^/H^+^ exchangers is shown. Residues that coordinate the ion in PaNhaP are marked with a *diamond*. The crystal structure of PaNhaP (Protein Data Bank code 4CZA) was reported in Ref. 5.

The X-ray structure of PaNhaP shows that the two acidic side chains of Asp-159 and Glu-73 are directly involved in binding of the Tl^+^ ion, whereas Asp-130 coordinates the ion via a water molecule (Fig. 5). Our electrophysiological measurements are in agreement with this finding. A comparison with the putative Na^+^ binding site of MjNhaP1 is instructive. MjNhaP1 and related transporters lack Glu-73 (see sequence comparison in Fig. 5) and have a neutral side chain in this position. Compared with MjNhaP1, the Na^+^ binding activity of PaNhaP extends into the acidic pH range (Fig. 1) and the pH-dependent profile (Fig. 2*A*) decreases roughly linearly with pH. This would not be expected in a typical titration curve. When Glu-73 is removed, the pH profile of PaNhaP resembles that of MjNhaP1, which has an uncharged side chain in this position (Fig. 2*C*). This demonstrates that Glu-73 is responsible for the extended Na^+^ binding pH profile of PaNhaP. At the same time, the Na^+^ affinity of PaNhaP_E73A_ is reduced to 10%, most likely due to weaker coordination of the substrate ion.

**FIGURE 6.**
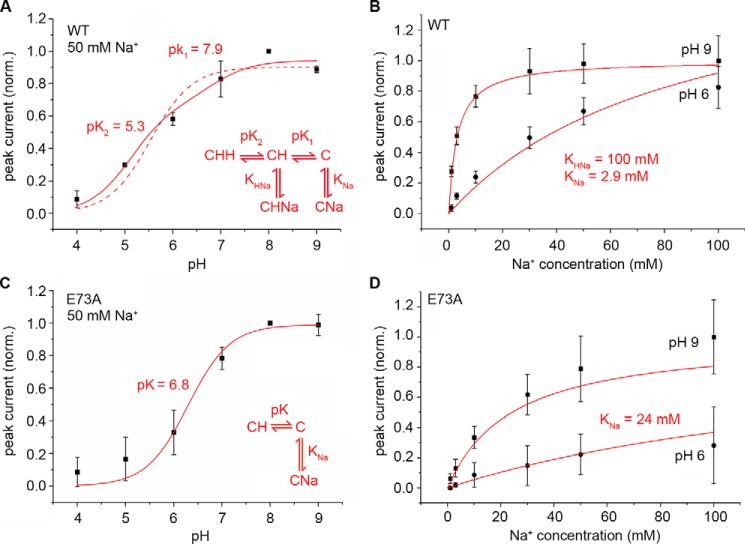
**Model calculation of currents at different pH and Na concentration.** Experimental data from Fig. 2 and model calculations of currents at different pH and Na^+^ concentrations using the kinetic model given in the figure and described in the text. *Red lines* represent fits of the pH dependences of PaNhaP_WT_ (*A*) and PaNhaP_E73A_ (*C*) with 50 mm Na^+^ concentration jumps or for the Na^+^ dependences of the PaNhaP_WT_ (*B*) and PaNhaP_E73A_ (*D*) at pH 6 and 9. Kinetic parameters used for the model calculation are given in the figure. The *dashed line* in *A* demonstrates the poor fit obtained with a single p*K* titration curve.

Further support for this notion comes from the kinetic analysis. Because the observed transient currents represent Na^+^ binding, we analyzed the pH profile of PaNhaP_WT_ in terms of a hypothetical kinetic model, which includes two H^+^ binding sites representing the Glu-73 and Asp-159 residues that directly coordinate the Na^+^ ion. Here we made the simplifying assumption that the same amount of charge is displaced when Na^+^ binds to the unprotonated or the singly protonated binding site (see kinetic model in Fig. 6*A*). A fit with the experimentally determined Na^+^ binding constants of 2.9 and 31 mm, respectively (Table 1), reproduces the pH profile of PaNhaP very well, yielding p*K* values 7.9 and 5.3 due to the two acidic side chains (Fig. 6*A*). Accordingly, the pH dependence of PaNhaP_E73A_ can be fitted with a single p*K* of 6.8, which resembles that of MjNhaP1 (7). Note that this kinetic model not only agrees with the pH profile but also with the Na^+^ dependence of PaNhaP_WT_ and PaNhaP_E73A_ (Fig. 6, *B* and *D*).

In conclusion, functional measurements with Na^+^ as substrate cation are in close agreement with Tl^+^ binding observed in the crystal structure and show that two carboxylates, Glu-73 and Asp-159, are directly involved in binding the substrate ion. Furthermore, our measurements show that the additional carboxylate in the Na^+^ binding site of PaNhaP significantly increases the Na^+^ affinity and extends its Na^+^ binding capacity to the acidic pH range. This is most probably also the reason why the active pH range of PaNhaP is shifted to acidic pH (5) compared with MjNhaP1 (4).

##### Tl^+^ Binds to the Na^+^ Binding Site of PaNhaP with Low Affinity

It has previously been shown that Tl^+^ is transported by PaNhaP (5). Likewise, transient currents were also obtained with Tl^+^ instead of Na^+^ (Fig. 4*A*). Furthermore, the experiment in Fig. 4*B* shows that Na^+^ and Tl^+^ compete for the same binding site. We conclude that the Tl^+^ binding site in the X-ray structure is the same as that for Na^+^.

There is a significant difference between Na^+^- and Tl^+^-induced currents. The amplitude of the Tl^+^ currents is roughly 10 times smaller than that of Na^+^ currents and the affinity of the PaNhaP cation binding site for Tl^+^ is 10-fold lower than for Na^+^. Both results indicate that the Tl^+^-bound state is different from the Na^+^-bound state. This may indicate incomplete occlusion of Tl^+^. Alternatively, Na^+^ binding may permit subsequent conformational transitions that could contribute to charge displacement that cannot take place in the Tl^+^-bound state.

##### Physiological Role of Microbial Na^+^/H^+^ Exchangers

**FIGURE 7.**
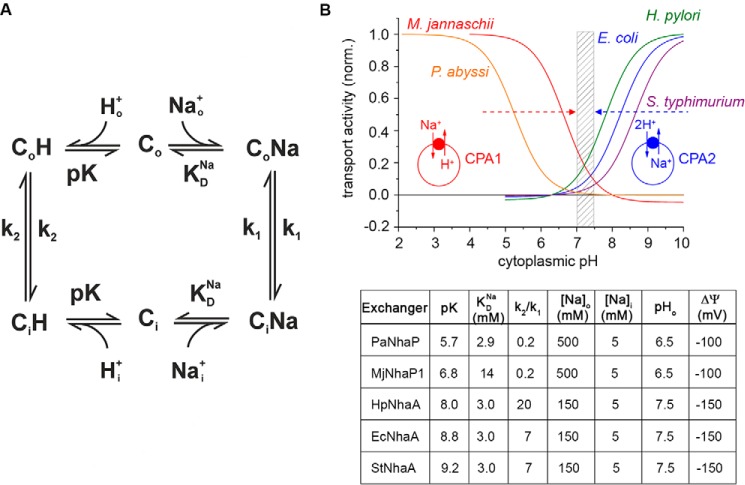
**Kinetic model and calculated pH profiles of the transport activity of microbial CPA1 and CPA2 Na^+^/H^+^ exchangers under physiological pH conditions.** *A*, kinetic model of Na^+^/H^+^ exchange. *B*, pH profiles calculated according to the kinetic model shown in *A*. Kinetic parameters and environmental conditions used for the calculation are given in the *lower panel* taken from this and previous publications (see ”Experimental Procedures“ for references). Typical values for cytoplasmic and extracellular Na^+^ concentrations and extracellular pH were chosen according to the natural habitat of the host organisms. (*EcNhaA*, NhaA Na^+^/H^+^ exchanger of *E. coli*; *HpNhaA*, NhaA Na^+^/H^+^ exchanger of *H. pylori*; *MjNhaP1*, NhaP1 Na^+^/H^+^ exchanger of *M. jannaschii*; *PaNhaP*, NhaP Na^+^/H^+^ exchanger of *P. abyssi*; *StNhaA*, NhaA Na^+^/H^+^ exchanger of *S. typhimurium.*)

Using the kinetic model given in Fig. 7 we can calculate the activity profile of the PaNhaP_WT_ Na^+^/H^+^ exchanger at physiological conditions as a function of the cytoplasmic pH from the kinetic parameters in Table 1. The p*K* of the binding site was approximated by the experimentally determined apparent p*K*_app_ and the Na^+^ binding constant by the experimentally determined *K_m,alk_*(Na^+^). This is a good approximation because, at the slow turnover rate of these exchangers, the transient currents correspond to electrogenic Na^+^ binding rather than transport. For comparison we also calculated the pH profile of MjNhaP1, another CPA1 Na^+^/H^+^ exchanger.

Both *P. abyssi* and *M. jannaschii* were isolated from hydrothermal vents and grow optimally under conditions of high salinity (13, 14). Therefore, the normal mode of transport for PaNhaP and MjNhaP1 will be import of Na^+^ and export of H^+^. This is the same as the role of the plasma membrane NHE eukaryotic exchangers that regulate Na^+^ import, coupled with alkalinization of the cell (15). pH regulation should be, indeed, a main concern of the extremophiles *P. abyssi* and *M. jannaschii*, as they can encounter rather severe pH changes in their natural environment as a result of mixing between the acidic hydrothermal fluids with the slightly alkaline seawater (16). As shown in Fig. 7*B*, the activity of CPA1 exchangers is maximal when the cytoplasmic pH drops below neutral and the transporter imports Na^+^ ions to increase the internal pH. Interestingly, the activity of PaNhaP is shifted toward the acidic range, which might indicate that *P. abyssi* is more prone to acidification than *M. jannaschii*, despite the fact that the optimum pH for growing *P. abyssi* is 6.8 (13), whereas it is 6 for *M. jannaschii* (14). An acidic shifted p*K* of PaNhaP would be in line with the metabolic difference between the two species. *P. abyssi* produces acidic compounds (including H_2_S) and CO_2_ as the end products of its metabolism (17), whereas *M. jannaschii* consumes CO_2_ in the process of methanogenesis (14, 18).

In theory, both CPA1 exchangers can also operate in reverse to export Na^+^ when the cytoplasmic pH shifts to the alkaline. However, under normal growth conditions, at an outside Na^+^ concentration of ∼0.5 m, this mode would be very slow because the Na^+^ concentration gradient that would have to be overcome is high. By comparison, the three NhaA CPA2 exchangers shown in Fig. 7*B* export Na^+^ against high concentration gradients by virtue of their much higher p*K* (thus favoring periplasmic H^+^ binding). Moreover, they are electrogenic (2 H^+^ imported for one Na^+^ ion exported), so that one net positive charge is imported into the cell, which is favored by the overall negative-inside membrane potential.

In conclusion, a comparison of the investigated CPA1 and CPA2 Na^+^/H^+^ exchangers reveals an interesting self-regulating feature of both transporter families. The p*K* values of the different exchangers are carefully tuned to values that lead to down-regulation at neutral pH. Therefore, their activity would always drive the cytoplasmic pH into the desired neutral pH range (shown by the *dashed arrows* in Fig. 7). Note that this is true for CPA1 and CPA2 exchangers, even though they transport ions in opposite directions under physiological conditions. Once a neutral internal pH has been reached, they are inactive and no longer pose the risk of pH changes or excessive loss or accumulation of Na^+^.

## Experimental Procedures

### 

#### 

##### Protein Expression, Purification, and Reconstitution

The synthetic gene for wild-type PaNhaP (PaNhaP_WT_) was cloned with a C-terminal cysteine protease domain fusion into the pET21a plasmid. The PaNhaP mutation Glu-73 to Ala (PaNhaP_E73A_) was introduced by site-directed mutagenesis (19). The resulting plasmids were used to transform *E. coli* C41-(DE3) cells and target proteins were purified as described (5). Purified proteins were reconstituted at a lipid to protein ratio (w/w) of 4 into liposomes prepared from *E. coli* polar lipids (Avanti Polar Lipids, Inc., Alabaster, AL) as described (5).

##### SSM-based Electrophysiology

Electrophysiological measurements were performed essentially as described (7). Briefly, 30 μl of proteoliposome suspension were allowed to adsorb for at least 1 h to the SSM sensor on which an octadecanethiol/phospholipid hybrid bilayer had been formed. A single-solution exchange protocol was employed, in which non-activating or activating solutions flowed successively for 0.5 s over the SSM membrane.

Solutions used for Na^+^ concentration jumps containing 50 mm MES, 50 mm HEPES, 50 mm Tris, 200 mm choline chloride, 5 mm MgCl_2_, and 1 mm dithiothreitol were titrated to the desired pH with Tris or HCl. In addition, non-activating solutions contained 100 mm choline chloride, whereas activating solutions contained *x* mm NaCl and (100 − *x*) mm choline chloride. Most concentration jump experiments were performed with a single-solution exchange protocol of the form: non-activating/activating/non-activating solution (20), where the pH on both sides of the proteoliposome membrane was identical (symmetrical pH). For the double-solution exchange flow protocol (20) the pH was varied independently inside and outside the proteoliposomes (asymmetrical pH). This required an additional resting solution, which was identical to the activation solution except for the pH (21).

Solutions to assess the inhibitory effect of Tl^+^ on Na^+^ concentration jumps contained 50 mm MES, 50 mm HEPES, 50 mm Tris, 200 mm KCH_3_COO, 5 mm Mg(CH_3_COO)_2_. Non-activating solutions contained in addition 100 mm KCH_3_COO, whereas activating solutions contained *x* mm NaCH_3_COO and (100 − *x*) mm KCH_3_COO. All solutions were supplemented with various concentrations (0–150 mm) of TlCH_3_COO. Tl^+^ measurements were performed at symmetrical pH 8.

Solutions to assess the effect of Tl^+^ concentration jumps contained 50 mm MES, 50 mm HEPES, 50 mm Tris, 200 mm KCH_3_COO, 5 mm Mg(CH_3_COO)_2_. Non-activating solutions contained either an additional 100 mm KCH_3_COO or 50 mm NaCH_3_COO + 50 mm KCH_3_COO, whereas activating solutions contained 30 mm TlCH_3_COO and an additional 70 mm KCH_3_COO or 30 mm TlCH_3_COO + 20 mm KCH_3_COO + 50 mm NaCH_3_COO.

Transient currents were also recorded for Na^+^ concentration jumps performed on empty liposomes that did not contain the reconstituted transporter. For Na^+^ concentration jumps, the amplitude of these currents was subtracted from the amplitude of currents measured on proteoliposomes.

##### Counterflow Assay

For counterflow measurements PaNhaP_WT_was reconstituted as described (5) with the following modification. The reconstitution buffer contained 10 mm tricholine citrate/Tris and 10 mm NaCl. Liposomes were diluted 1:100 in activity buffer (10 mm tricholine citrate/Tris, 2 mm MgSO_4_, 20 μm carbonyl cyanide *m*-chlorophenylhydrazone, 1 μCi/ml of ^22^Na) that was supplemented with NaCl to a final concentration of 500 μm. 200 μl of the sample was filtered on 0.22-μm nitrocellulose filters (GSWP02500, Millipore) that were subsequently washed with 3 ml of ^22^Na-free activity buffer. Filters were transferred to vials and overlaid with 4 ml of liquid scintillation mixture (Rotiszint, Carl Roth, Karlsruhe, Germany) for counting. Measurements were carried out at room temperature at pH 6 and 8 (same inside and outside pH). Empty liposomes were used as a control.

##### Kinetic Analysis

For the kinetic models (Figs. 6 and 7) steady state analytical solutions were calculated using the software Mathcad (Parametric Technology Corp., Needham, MA). For the analytical solution of the kinetic model of Fig. 7, see Ref. 6.

To investigate the role of Na^+^/H^+^ exchangers under physiological conditions, the transport activity was calculated using a kinetic model (Fig. 7*A*) and experimentally determined kinetic parameters. The relevant parameters for the calculation are compiled in Fig. 7. For the CPA2 NhaA-type Na^+^/H^+^ exchangers of *Helicobacter pylori*, *E. coli*, and *Salmonella typhimurium*, previously determined kinetic parameters were used (10). For the CPA1 NhaP-type Na^+^/H^+^ exchangers kinetic parameters were taken from Table 1 for PaNhaP_WT_ and Ref. 7 for MjNhaP1.

For bacteria of the gastrointestinal tract (*H. pylori, E. coli*, and *S. typhimurium*) a Na^+^ concentration of 150 mm and pH 7.5 were taken as typical environmental conditions. The environment of *H. pylori* can be highly acidic. However, it was shown that the pH at the plasma membrane is maintained close to neutral pH by the urease system (22). The archaea *M. jannaschii* and *P. abyssi* colonize submarine hydrothermal vents where pH is typically slightly acidic. We assumed pH 6.5, which is an average value of optimal growth pH for *M. jannaschii* (14) and *P. abyssi* (13), and the Na^+^ concentration of sea water, which is ∼0.5 m.

## Author Contributions

K. F. initiated and directed the project. O. C., M. L., and D. W. performed the experiments described. K. F., W. K., Ö. Y., and O. C. analyzed the data and wrote the manuscript. All authors reviewed the results and approved the final version of the manuscript.

## Footnotes

2 The abbreviations used are:

CPA: cation proton antiporter
EcNhaA: NhaA Na^+^/H^+^ exchanger of *E. coli*
MjNhaP1: NhaP1 Na^+^/H^+^ exchanger of *M. jannaschii*
NHE: mammalian Na^+^/H^+^ exchanger
PaNhaP: NhaP Na^+^/H^+^ exchanger of *P. abyssi*
SSM: solid supported membrane.
